# Listening in the Mix: Lead Vocals Robustly Attract Auditory Attention in Popular Music

**DOI:** 10.3389/fpsyg.2021.769663

**Published:** 2021-12-23

**Authors:** Michel Bürgel, Lorenzo Picinali, Kai Siedenburg

**Affiliations:** ^1^Department of Medical Physics and Acoustics, University of Oldenburg, Oldenburg, Germany; ^2^Dyson School of Design Engineering, Imperial College London, London, United Kingdom

**Keywords:** auditory attention, polyphonic music, singing voice, vocal salience, music mixing

## Abstract

Listeners can attend to and track instruments or singing voices in complex musical mixtures, even though the acoustical energy of sounds from individual instruments may overlap in time and frequency. In popular music, lead vocals are often accompanied by sound mixtures from a variety of instruments, such as drums, bass, keyboards, and guitars. However, little is known about how the perceptual organization of such musical scenes is affected by selective attention, and which acoustic features play the most important role. To investigate these questions, we explored the role of auditory attention in a realistic musical scenario. We conducted three online experiments in which participants detected single cued instruments or voices in multi-track musical mixtures. Stimuli consisted of 2-s multi-track excerpts of popular music. In one condition, the target cue preceded the mixture, allowing listeners to selectively attend to the target. In another condition, the target was presented after the mixture, requiring a more “global” mode of listening. Performance differences between these two conditions were interpreted as effects of selective attention. In Experiment 1, results showed that detection performance was generally dependent on the target’s instrument category, but listeners were more accurate when the target was presented prior to the mixture rather than the opposite. Lead vocals appeared to be nearly unaffected by this change in presentation order and achieved the highest accuracy compared with the other instruments, which suggested a particular salience of vocal signals in musical mixtures. In Experiment 2, filtering was used to avoid potential spectral masking of target sounds. Although detection accuracy increased for all instruments, a similar pattern of results was observed regarding the instrument-specific differences between presentation orders. In Experiment 3, adjusting the sound level differences between the targets reduced the effect of presentation order, but did not affect the differences between instruments. While both acoustic manipulations facilitated the detection of targets, vocal signals remained particularly salient, which suggest that the manipulated features did not contribute to vocal salience. These findings demonstrate that lead vocals serve as robust attractor points of auditory attention regardless of the manipulation of low-level acoustical cues.

## Introduction

In everyday life, our sense of hearing is exposed to complex acoustical scenes that need to be analyzed and interpreted. The ability to segregate an acoustic scene into a mental representation of individual streams is known as auditory scene analysis (ASA; Bregman and McAdams, 1994). A prime example of this is listening to music with multiple instruments playing at once. Human listeners can focus and track a single instrument remarkably well, even though the acoustic signal is a potentially ambiguous clutter of diverse instrument signals.

Two interwoven analytical processes are used in ASA: endogenous top-down and exogenous bottom-up processes. Endogenous processes are based on cortical functions, such as expectations, learned patterns, and volition. Exogenous processes are driven by pre-attentive processes based on the temporal and spectral properties of a sound, from which auditory attributes, such as duration, pitch, or timbre are computed, and which are pivotal for grouping auditory information into separate sound events. Timbre, often simply described as “texture” or “tone color” (Helmholtz, 1885), is a multidimensional attribute (Siedenburg and McAdams, 2017) that enables the discrimination of sound sources (e.g., sounds from a keyboard vs. a guitar), even though they may match in other acoustic cues such as loudness and pitch.

A well-established approach to the study of ASA and auditory attention is the use elementary auditory tasks, such as the presentation of sequential or simultaneous streams of tones (for a review, see Alain and Bernstein, 2015). Bey and McAdams (2002) investigated the influence of selective attention in ASA using two-tone sequences, one of which was interleaved with distractor tones. The semitone spacing between the distractor tones and the target sequence was varied from 0 to 24 semitones, thereby varying the strength of exogenous cues that allow for bottom-up stream segregation. Participants had to judge whether the sequences were different or identical and had to ignore the distractors. To vary the dependency on selective attention, in one condition, the stream with distractor tones was presented first, followed by the melody without distractors; in a second condition, selective attention was facilitated by presenting the melodies without distractors first, thus providing a pattern that could be compared with the following mixture. The results showed that participants achieved higher recognition rates when the melodies without distractors were presented first, thus being able to selectively attend to the target melody.

Another more ecological approach uses polyphonic music to study ASA. In polyphonic music, multiple relatively independent melodies (also referred to as voices) are played or sung simultaneously. Behavioral studies showed that when listening to polyphonic music a superior perception of timing and meter is found in the lower voices (Hove et al., 2014), whereas tonal and melodic perception is facilitated in the highest voice (Crawley et al., 2002). Accordingly, the so-called high-voice superiority effect states that the voice with the highest pitch trajectory is most salient in polyphonic mixtures (Fujioka et al., 2005). It has been shown that this effect is present in infants (Marie and Trainor, 2013) and that it can be enhanced by musical training (Marie et al., 2012). Using a model of peripheral auditory processing, results by Trainor et al. (2014) suggest that the origin of high-voice superiority may be based on physiological factors such as cochlear filtering and masking patterns.

Another factor that has been shown to affect musical scene perception and the specific trajectory of auditory attention is related to the repetitiveness of musical voices. Taher et al. (2016) found that when a repetitive and non-repetitive voice is playing simultaneously, attention is drawn to the non-repetitive voice. Barrett et al. (2021) investigated whether the coherent timings between instruments in a piece of music facilitate stream segregation. The authors either slowed down one instrument or recomposed an instrumental line so that it no longer matched with the other lines. The results suggested that, when instruments are temporally coherent, attention is not directed to a particular instrument, and therefore instruments are integrated into one percept. For incoherent musical lines, attention was drawn toward one instrument while the other instrument was ignored. A study by Disbergen et al. (2018) focused on the effect of timbre dissimilarity for distinguishing between two melodic voices in polyphonic music. Although no clear effect for a modification of timbral dissimilarity could be observed, the results implied a trend that a reduction of timbral dissimilarity and thus a reduction of acoustical cues lead to a deterioration of stream segregation, further suggesting that a minimum of exogenous cues is necessary to track and separate single streams. In Siedenburg et al. (2020), listeners had to hear out instruments and melodies of varying sound level masked by a simultaneously playing instrument. It was found that participants were able to exploit dips in the masker signal, allowing them to hear the target instrument at lower levels than with a masker that did not contain these dips.

Several of the aforementioned studies used (simplified or stylized) excerpts of Western classical instrumental music. In Western popular music, the lead melody and thus the centerpiece of a song is sung by a human voice (lead vocals), which is accompanied by a variety of instruments and, at times, background vocals. Recent studies have shown that the voice occupies a unique role among other sound sources (e.g., Belin et al., 2000; Levy et al., 2001; Agus et al., 2012; Suied et al., 2014; Isnard et al., 2019). In a neurophysiological study, Belin et al. (2000) examined the response to speech, vocal non-speech sounds, and non-vocal environmental sounds. The data implied not only that cortical activity to vocal speech and non-speech sounds were higher than to non-vocal environmental sounds but also that specific regions in the human cortex responded more strongly to vocal sounds, suggesting a specialized processing of speech sounds. Levy et al. (2001) measured neurophysiological data from participants in an oddball task in which single instruments and singing voice were presented sequentially. A piano sound was used as a target, while other sounds were used as distractors. The results showed a stronger response to the presentation of the human voice, termed the “voice-specific response.” The authors hypothesized that this response represented a gating mechanism in which the auditory system allocates the input to be processed phonologically. In Agus et al. (2012), accuracy and reaction times were investigated in a sound classification task. Single notes were played by instruments, sung by voices, or played by interpolations between instruments and voices (i.e., chimeras). Accuracy for voices was higher and reaction times were faster than for all other target categories, indicating an advantage in processing voices. Studies by Suied et al. (2014) and Isnard et al. (2019) focused on the recognition of timbre in short glimpses of recorded sounds that differed only in timbre. Again, singing voices stood out by achieving recognition above chance level with a sound duration of only 4 ms, while all other instrument categories required 8 ms durations.

In the present study, we aim to investigate auditory attention in an instrument and singing voice detection task inspired by everyday music listening of popular music. To study how the detection of different instruments is modulated by auditory attention, we vary the presentation order of mixture and target cue. In one order of presentation, a cue from a target vocals or instrument is presented first, followed by the mixture, such that the cue can be used to search the mixture for the target. In the reverse presentation order, the mixture is presented first followed by the target cue. Based on experiments such as Bey and McAdams (2002), we expect that the order in which a cue is presented first facilitates detection of the target. Motivated by the distinct role of singing voices that has been reported in the literature, we investigated whether the lead vocals in popular music would play a special role in auditory scene analysis (ASA) and selective listening. Based on this assumption, we hypothesize that lead vocals achieve distinctly higher accuracies in both presentation orders.

## General Methods

**Figure 1:**
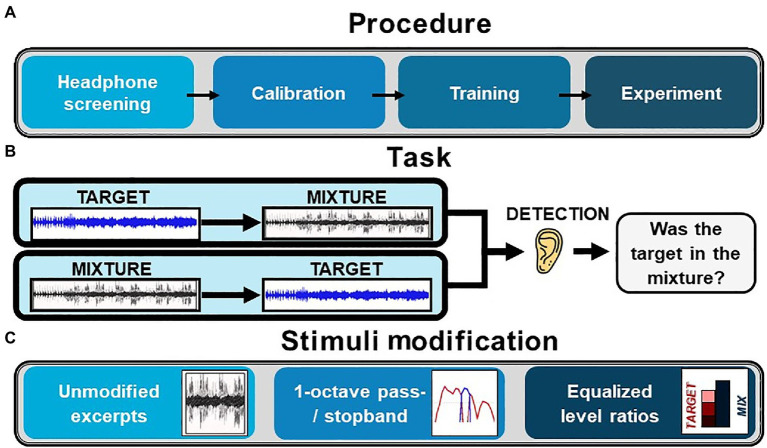
Schematic overview of the experiments. **(A)** Procedure: The experiment started with a headphone screening task, followed by a subjective sound level calibration, a training section where participants were familiarized with the instrument detection task and finally the main experimental section. **(B)** Task: An instrument detection task was used in the experiments: Participants either took part in an experiment where the targets were preceding the mixtures or where the mixtures were preceding the targets. **(C)** Stimuli modification: In the first experiment, excerpts unmodified from their original state were used. In the second experiment, the targets were filtered in an octave band to create a spectral region in which the target could pass without being spectrally masked. In the third experiment, the individual sound level differences between the diverse vocals and instruments were adjusted to one of three possible level ratios.

For our experiment, we used short excerpts of popular music in which either a cued target instrument or target vocal was present or absent in a mixture of multiple instruments (see Figure 1B). To test the effects of selective auditory attention, we interchanged the presentation order of the cue and mixture (Bey and McAdams, 2002). This yielded two different listening scenarios: one requiring selective listening, and the other requiring a rather global mode of listening. When the target was presented prior to the mixture, selective attention could be used to detect the target in the mixture. When the target was presented after the mixture, listeners had to be aware of possibly all components of the mixture and hence listen more globally to the excerpts. In that case, attention could be affected by exogenous factors, for instance the salience of individual sounds in the musical scene. We conducted three experiments aimed to study the role of attention in the processing of popular music mixtures and whether acoustic modifications of the excerpts would manipulate the detection of instruments or vocals. For the first experiment, we left the excerpts unmodified and investigated the detection accuracy in the complex musical scene and how it was affected by the presentation order and different instruments. In the second experiment, we aimed to suppress energetic masking of the target by means of bandpass/bandstop-filtering. To control the influence of instrument dependent sound levels, we equalized the sound levels ratios between the different targets in the third experiment. A schematic overview of the experiments is shown in Figure 1C. The same general methods were applied in all three experiments. Specific modifications of the methods are described in detail in the respective experiments (see Unmodified Excerpts, Spectral Unmasking Equalization, and Sound Level Equalization).

### Participants

All participants were students recruited *via* an online call for participation at the e-learning platform of the University of Oldenburg. General information about the experiment and exclusions criteria were given. The criteria included the use of headphones, a stable internet connection, and self-reported normal hearing. Participants could start the online experiment at any time *via* a link that was provided in a personalized email. Participation was compensated monetarily. We acquired information about the participants musical training using five questions: Number of instruments played, hours practiced during the period of greatest musical interest, years of lessons in music theory, years of lessons for an instrument, and self-designation (non-musician, amateur musician, and professional musician).

### Stimuli and Task

**Figure 2:**
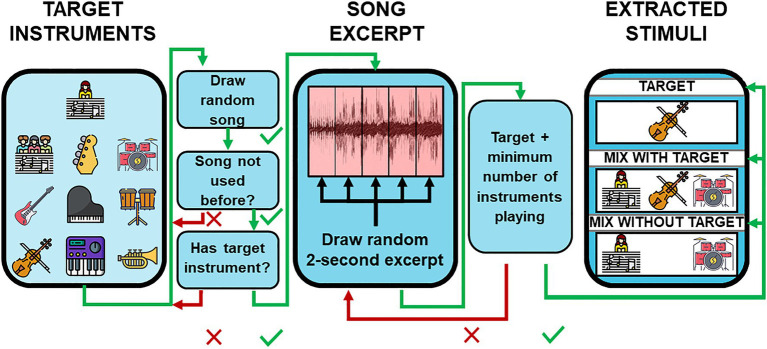
Stimuli extraction. Short excerpts from a multitrack database containing reproductions of popular music were used as stimuli. The schematic shows the workflow of the stimulus construction. For details, see the text.

An illustration of the stimuli extraction is shown in Figure 2. Stimuli were generated using a Matlab script (MathWorks Inc., Natick, MA, United States) that extracted 2-s excerpts from a multitrack music database. The database was created by Tency Music and is used within the Musiclarity web-app (Eastgate et al., 2016). It consists of sound alike reproductions of well-known popular music with English lyrics and individual audio files for each instrument. The Instruments in the database were coarsely categorized as: Backing Vocals, Bass, Drums, Guitars, Lead Vocals, Piano, Percussion, Strings, Synthesizer, and Winds. For each excerpt, one to-be attended instrument was chosen (target). Other instruments in the excerpt that were not from the same category as the target served as maskers (mixture). Instruments from the same category that were not used as a target were excluded from the mixture. When lead vocals were assigned as the target, all backing vocals were also excluded. Songs were drawn pseudo-randomly, with the same song chosen as infrequent as possible. To investigate which instruments were audible at any given time, the sound level of each instrument was analyzed using a 500 ms sliding window. In each window, the root-mean-squared (RMS) sound level was calculated. Windows were qualified as potential candidates for the excerpt extraction if one instrument in the target category and 6–9 additional instruments had sound levels above −20 dB relative to the instrument’s maximum sound level across the full song. A previously unused 2,000 ms time slice containing four qualified adjacent 500 ms windows was randomly drawn. Three monophonic signals were compiled from each 2-s excerpts: (1) a signal only containing the target, (2) a signal containing a mixture of 5–8 instruments from non-target categories plus the target. (3) A signal containing a mixture of 6–9 instruments without the target. For mixtures, the full number of instruments was used, which were also present in the original excerpt of the song. A logarithmic fade-in and fade-out with duration of 200 ms was applied to the beginning and end of all extracted signals. For half of the trials, the mixture signals were arranged to contain the target signals, and for the other half, the mixture did not contain the target signal. From these signal combinations, two stimuli with duration of 4,500 ms were created using different presentation orders for the target and mixture signal. In the “Target-Mixture” condition, the target signal was followed by a 500 ms pause and the mixture signal; in the “Mixture-Target” condition, the presentation order was reversed. For the use on the online platform, the stimuli were converted from WAV format to MP3 with a bit rate of 320 kbit/s. Example stimuli are provided on our website: https://uol.de/en/musik-wahrnehmung/sound-examples/listening-in-the-mix

### Procedure

The experiments were approved by the ethics board of the University of Oldenburg and carried out online *via* the web platform www.testable.org. Participants were divided into one of two groups. For group 1, all stimuli had the presentation order “Target-Mixture,” whereas for group 2 the presentation order was reversed (see Figure 1B). The same excerpts were used for both groups, thus the only differences were in the order of presentation. Each experiment was further divided into four consecutive segments (see Figure 1A).

At the beginning of the experiment, participants had to fill in a form regarding personal data (see General Methods). To get an indication of whether participants were using headphones, a headphone screening task was performed at the beginning of the experiments. For Experiment 1 headphone screening was based on Woods et al. (2017), employing a sequential presentation of three pure tones, where one of the tones was quieter. The tones were phase shifted on the left side by 180° and therefore appeared attenuated when listening over loudspeakers but not attenuated when headphones were worn. Therefore, a matching volume judgment should only be achieved by wearing headphones. Listeners had to detect the quiet tone and passed the test if five out of six detections were correct. For Experiments 2 and 3, the headphone screening was based on Milne et al. (2020), which provides a higher selectivity for headphone users than the headphone screening used in Experiment 1. Here, a sequence of three white noise signals were presented, where one of the noise signals was phase shifted by 180 degrees in a narrow frequency band at around 600 Hz on the left headphone channel. When headphones were worn, the phase shift was perceived as a narrow tone embedded in the broadband noise. Listeners had to detect the tone and passed the test if five out of six detections were correct. Participants who failed the headphone screening were removed from the data analysis.

After the headphone screening, three song excerpts were presented aiming to provide an impression of the dynamic range of the stimuli. During the presentation, participants were instructed to adjust the sound to a comfortable level. This was followed by a training phase, where participants were familiarized with the detection task. Participants listened to stimuli akin to those used in the main experiment and were asked whether the target was present or absent in the mixture. For each category, one stimulus with and without target was presented. To help participants understand the task and to make them more sensitized for the acoustic scene, feedback was given after each answer. This was followed by the main experiment where the same procedure was used but no feedback was given. Stimuli presented in the training segment were not reused in the experiment segment. All stimuli were presented in a random sequence that intermixed all conditions (except for the between-subjects factor of presentation order). The number of stimuli, the conditions, and the target categories differed from experiment to experiment and are therefore described in the sections on the individual experiments below.

### Data Analysis

Following the methodology recommended by the American Statistical Association (Wasserstein et al., 2019), we refrain from the assignment of binary labels of significance or non-significance depending on an immutable probability threshold. We provide mean detection accuracies, followed by a square bracket containing the 95% CIs computed by means of bootstrapping and round brackets containing the decrease or increase through a change in presentation order.

A generalized binominal mixed-effect model (West et al., 2014) was used for the statistical analysis. All mixed-effects analyses were computed with the software R (R Core Team, 2014) using the packages lme4 (Bates et al., 2015), which was also used to estimate marginal means and CIs. Our model included random intercepts for each participant and item (i.e., stimulus). All binary categorical predictors were sum-coded. The correlation coefficients of the model are given as standardized coefficients (*χ*^2^) and probability (p). To summarize the main effects and interactions, results are presented in the form of an ANOVA table, derived from the GLME models *via* the *anova* function from the car package (Fox and Weisberg, 2019). A detailed view of the behavioral results, models and statistic evaluations for each experiment are presented in the supplementary material (see Supplementary Tables 1–6).

### Method Validation

Since the experiment was conducted online, and therefore did not undergo the strict controls of a laboratory experiment, we compared results for using calibrated laboratory equipment and consumer devices. In order to achieve this, a pilot experiment that was very similar to Experiment 1 was completed by the members of the Oldenburg research lab. In one condition, participants used their own computer and headphones. In another condition, they used calibrated audio equipment, and the presentation order of these two conditions was counterbalanced across participants. The calibrated equipment consisted of a laptop, RME Babyface soundcard, and Sennheiser HD650 headphones. The long-term sound level was set to 75 dB SPL (A), measured with Norsonic Nor140 sound-level meter using music-shaped noise as the excitation signal. Results showed very similar data for both types of equipment (for details, see Supplementary Figure 1), which did not indicate any systematic problem in conducting the present study *via* online experiments.

## Experiment 1: Unmodified Excerpts

The first experiment was our starting point to investigate selective auditory attention in musical scenes. We left the excerpts in their original state (as described in General Methods). As target categories besides the lead vocals, we chose four instrument categories that had shown rather diverse results in a pilot experiment.

### Participants

A total of 84 participants with a mean age of 25.1 years (SD = 4.5, range = 19–44) were tested in the experiment. A total of 25 out of 42 participants passed the headphone screening for the Target-Mixture condition and 22 out of 42 for the Mixture-Target condition (age = 25.3, SD = 5, range = 19–44). Only participants passing the headphone screening were included in further analysis. Eleven participants in the Target-Mixture condition and 10 participants in the Mixture-Target condition described themselves as either amateur or professional musicians.

### Stimuli and Procedure

For the first experiment, the following five target categories were selected: lead vocals, bass, synthesizer, piano, and drums. Headphone screening was based on Woods et al. (2017). In the training phase of the main experiment, one excerpt with a target and one excerpt without a target were presented for each of the five target categories, summing up to 10 stimuli in total. In the experimental phase, 150 stimuli were presented, divided into 30 stimuli for each of the five target categories. The average duration of the experiment was 25 min.

### Results and Discussion

**Figure 3:**
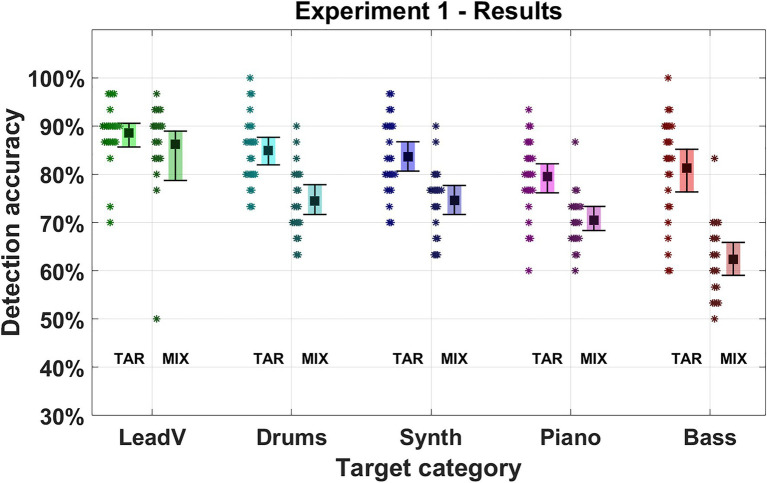
Detection accuracy in Experiment 1. Five instrument and vocal categories were used as targets (lead vocals, drums, synthesizer, piano, and bass). The Square marks the mean detection accuracy for a given target category. Error bars indicate 95% CIs. Asterisks represent the average accuracy of an individual participant for the given target category. “TAR” denotes the presentation order “Target-Mixture” where the target cue was presented followed by a mixture. “MIX” denotes the presentation order “Mixture-Target” where a mixture was presented followed by the target cue.

Figure 3 displays the average results of the first experiment for each instrument and presentation order (for numerical values, see Supplementary Tables 1, 2). Detection accuracy differed depending on the target category and order of presentation, which was also evident in our model (Instrument: *χ*^2^ = 97.881, *p* < 0.001, Order: *χ*^2^ = 38.878, *p* < 0.001.). Averaged across target categories, the Target-Mixture condition yielded the highest accuracy of 84% (70–97%), which deteriorated in the Mixture-Target condition to 72% (55–88%; −12%). This decline was strongest for the bass category in which the mean accuracy dropped by −19% from the Target-Mixture to the Mixture-Target condition. A nearly identical decrease was found for the synthesizers (−11%), piano (−8%), and drums (−11%). The lead vocals had the best performance overall and were least affected by a change in the presentation order (−2%). This resulted in an interaction effect between the instrument factor and the presentation order (*χ*^2^ = 13.059, *p* = 0.011).

All instruments except the lead vocals showed degraded detection accuracy when listeners were required to listen to the musical scenes without a cue. While the degradation of detection accuracy in a global listening scenario was to be expected (Bey and McAdams, 2002; Janata et al., 2002; Richards and Neff, 2004), the specific attentional bias towards lead vocals is, to our knowledge, a novel finding. We will refer to this unique characteristic as “lead vocal salience” in the following. This finding is in line with the unique role of singing voices documented in previous experiments, where voices were processed faster and more accurately in comparison to other musical instruments (e.g., Agus et al., 2012; Suied et al., 2014; Isnard et al., 2019) and were shown to have a unique cortical voice-specific-response indicating a specialized processing for human voices (e.g., Levy et al., 2001).

The bass was found to be most strongly affected by a change in presentation order, having medial detection accuracy in the Target-Mixture condition that, however, decreased almost 2-fold compared to the other instruments. One explanation for this could be tied to the spectral characteristics of the bass. The bass mostly occurs in a rather narrow band in the low frequencies, whereas other instruments cover a wider frequency range. When a cue is given, attention may be focused selectively toward that frequency band, and thus narrow signals like the bass can be reliably perceived. Another explanation could be derived from the high-voice superiority effect that has been observed in polyphonic music. The effect describes a pre-attentive attentional bias (Trainor et al., 2014), which, in the presence of multiple voices, draws attention toward the highest voices. In the current experiment, bass signals naturally correspond to low voices, and hence high-voice superiority may come into play.

It is to be noted, that our analysis revealed no systematic differences between participants who declared themselves as musician and those who did not. This held true across all three experiments, even though in previous studies, musicians showed improved results in ASA tasks (e.g., Başkent et al., 2018; Madsen et al., 2019; Siedenburg et al., 2020). The most likely reason to explain this may be that we did not specifically control for an equal number of musicians and non-musicians in a large sample; thus, the proportion of participants considered musicians were only a fraction of the total participants, and therefore the sample size may be too small for an adequate statistical comparison. We further analyzed how performance was affected by possible fatigue over the course of the experiment. Considering performance over the duration of the experiment averaged across subjects suggested that the difference between performance at the beginning and end of the experiment was negligible (for details, see Supplementary Figure 2).

**Figure 4:**
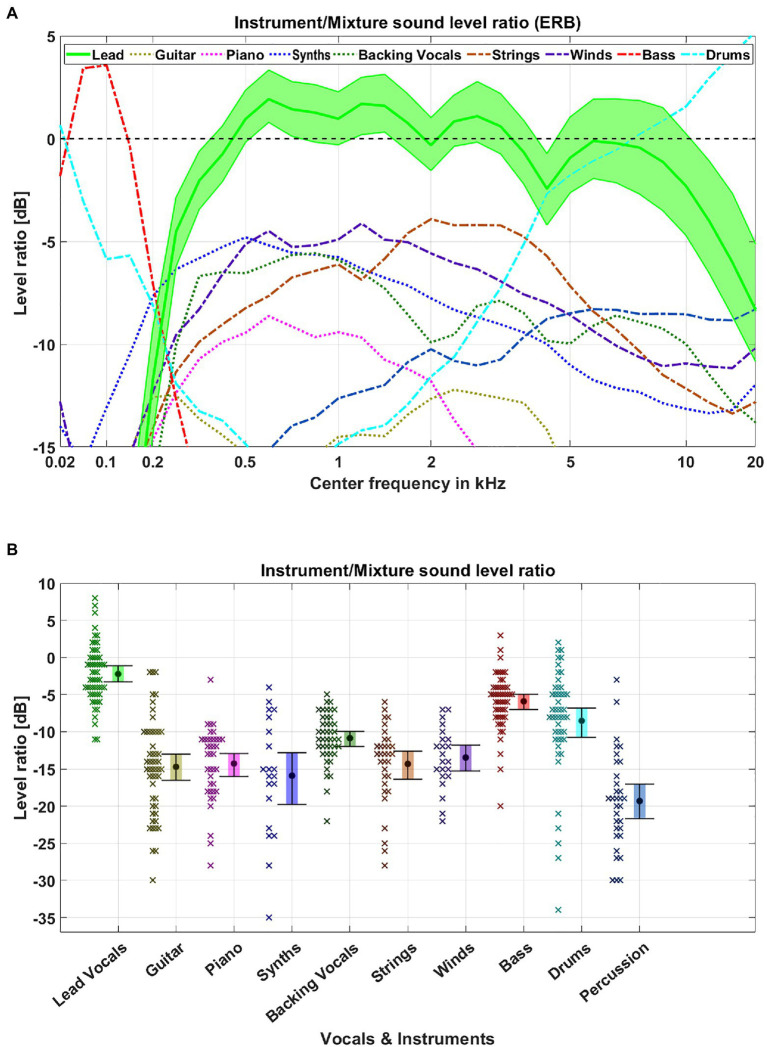
Database feature analysis. We analyzed the average sound level in ERB-bands **(A)** and broadband sound level **(B)** between each voice or instrument and the remaining mixture for each song. **(A)** Each colored line represents the average sound level for the given center frequency. The filled area represents the 95% CIs for the lead vocals. **(B)** The circle marks the mean detection accuracy for a given target category. Error bars indicate 95% CIs. Crosses represent the average level of an individual song for the given target category.

To further evaluate the acoustic origins of the lead vocal salience, we analyzed the music database in terms of spectral features and sound levels features. For each song and target category, we evaluated the broadband sound level as well as the sound level on an ERB-scale between all instruments and voices in a category and all other instruments and voices. We used a sliding window of 500 ms moving over the duration of a song and discarded all windows in which the sound level was less than 20 dB below the maximum sound level of the instruments, voices, or mixtures. The results of the time windows were then averaged for each song and are displayed in Figure 4.

The spectral analysis revealed a frequency region from 0.5 to 4 kHz, where the difference between the lead vocals and remaining mixtures had a positive level ratio (up to 2.5 dB), meaning that the lead vocals exhibited higher levels than the sum of accompaniment instruments and were therefore released from energetic masking in those spectral regions. While the lead vocals had a relative sound level of more than 0 dB in such a broad spectral region, only the bass and drums showed similar levels in either low or high frequencies. The other instruments did not have such a differentiated spectral range and their level was substantially below the level of the lead vocals. This was also evident in the broadband level analysis, in which the lead vocals had a significantly higher level than the other instruments. Accordingly, two acoustically-based explanations for the superior detection accuracy of the lead vocals could be (a) less susceptibility to masking by other instruments or (b) higher loudness levels of lead vocals. To scrutinize these two hypotheses, we conducted a second experiment where the vocals and the instruments were released from masking in the same frequency band, and a third experiment equalizing the sound level differences between lead vocals and instruments.

## Experiment 2: Spectral Unmasking Equalization

To investigate whether the observed lead vocal saliency was due to spectral masking, we here examined the spectral regions where vocals tended to be unmasked and applied the same unmasking to different target instruments. For this purpose, we analyzed the database for spectral regions in which the lead vocals exhibited particularly high sound levels. A broad spectral region from about 0.5 to 5 kHz was found. To provide equal masking and unmasking for all vocals and instruments, we used octave bands adjacent to the center of this region (1–2 and 2–4 kHz) and designed filters to pass signals only into one of the two bands (bandpass) or to suppress signals only into this range (bandstop). To compensate for level-dependent differences, the sound levels of all target instruments were adjusted identically. Only instruments with relevant intensity in the selected frequency bands were considered as targets for the experiment. Therefore, lead vocals, guitars, and piano were used as target categories. To avoid listeners focusing only on the octave bands, a randomly drawn accompaniment instrument was passed through the octave band for one third of trials, whereas the target category sound was attenuated in the octave band.

### Participants

A total of 49 participants with a mean age of 25.6 years (SD = 4.2, range: 20–39) were tested in the experiment. A total of 20 out of 25 participants passed the headphone screening for the Target-Mixture condition and 20 out of 24 or the Mixture-Target condition (age = 23.5, SD = 2.9, range: 20–29). Only participants passing the headphone screening were included in the analysis. Among these, 12 participants in the Target-Mixture condition and four participants in the Mixture-Target condition described themselves as either amateur or professional musicians.

### Stimuli and Procedure

In two out of three excerpts, the target was filtered through a passband either from 1 to 2 kHz or from 2 to 4 kHz, while the mixture was filtered through a bandstop in the same octave band. Excerpts filtered in this way are referred to as “TBP” in the following. To prevent participants to focus on only one of the two octave bands, in one third of the excerpts, a randomly drawn accompanying instrument was filtered through a passband of either 1–2 or 2–4 kHz, while the other accompaniment instruments and the target were filtered through a bandstop in the same octave band. Excerpts filtered this way are referred to as “TBS” in further analysis. Bandpass and bandstop filters were designed and applied using the corresponding Matlab functions *bandpass* and *bandstop* (Signal Processing Toolbox Release 8.3, MathWorks Inc., Natick, MA, United States). The filtered target signal was used both during the presentation of the cue and when it was presented in the mix. The signal components in the stopband were attenuated to −80 dB FS (decibels relative to full scale). Sound levels ratios between targets and mixtures were adjusted for all targets to −10 dB. In a final step, the average sound level of each stimulus was normalized to −15 dB FS. As target categories, lead vocals, guitar, and piano were chosen.

The headphone screening test was based on Milne et al. (2020). In the training phase of the main experiment, one stimulus with and one without a target were presented for each of the three target categories, each of the two octave bands and one additional stimulus for each target category and octave band where the target was filtered by a bandstop and an accompaniment instrument was filtered by a bandpass, summing up to 18 stimuli in total. In the experimental phase of the main experiment, 180 stimuli were presented, divided into groups of 60 for each of the three target categories and further subdivided into 20 stimuli for each octave band where the target was filtered by a bandpass plus 10 for each octave band where the target was filtered by a bandstop. The average duration of the experiment was 35 min.

### Results and Discussion

**Figure 5:**
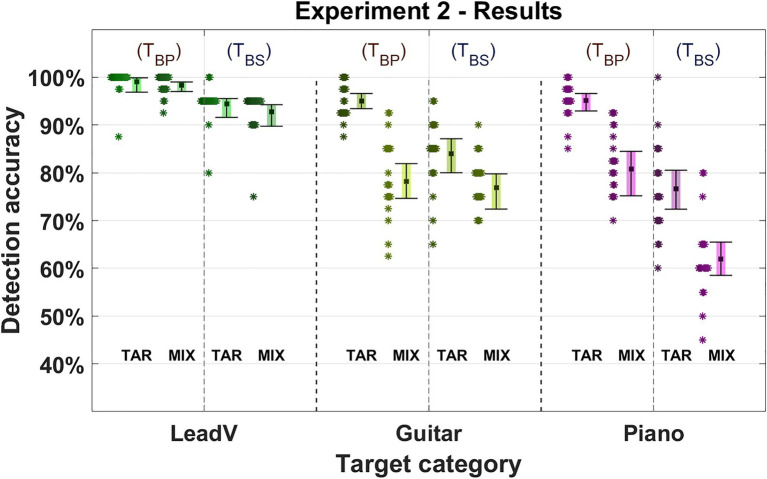
Detection accuracy in Experiment 2. Three instrument and vocal categories were used as targets (lead vocals, guitar, and piano). Either a bandpass or bandstop was applied to the filter and the mixture. The target filter type is listed in the upper area of the figure with T_BP_ indicating a bandpass was used and T_BS_ indicating a bandstop was used. The Square marks the mean detection accuracy for a given target category. Error bars indicate 95% CIs. Asterisks represent the average accuracy of an individual participant (*n* = 40) for the given target category. “TAR” denotes the presentation order “Target-Mixture” where the target cue was presented followed by a mixture. “MIX” denotes the presentation order “Mixture-Target” where a mixture was presented followed by the target cue.

Results are displayed in Figure 5 (for details, see Supplementary Tables 3, 4). Detection accuracy was affected by the filter type (TBP = target is filtered with a bandpass, TBS = target is filtered with bandstop), presentation order, and instrument type. While we used two different adjacent octave bands to filter the signals (1–2 and 2–4 kHz), results for both frequency bands showed nearly identical results with no systematic differences (differences for all conditions between both octave bands: Difference__MEAN_ = 2.5%, Difference__MIN_ = 1.5%, and Difference__MAX_ = 3.5%). This finding was underpinned by the GLME model, which revealed no effect for the usage of different octave bands (Octave: χ2 = 0.002, *p* = 0.963).

As in Experiment 1, the detection accuracy was better in the Target-Mixture condition and best when the target signal was filtered by a bandpass. The influence of both the order and the filter was reflected in our model (Order: *χ*^2^ = 3.547, *p* = 0.06, Filter: *χ*^2^ = 18.657, *p* < 0.001). For the Target-Mixture TBP condition, an average accuracy of 96% (95–97%) was observed compared to the 85% (82–89%; −11%) in the Mixture-Target condition. For the Target-Mixture TBS condition, an average accuracy of 85% (82–88%) was achieved compared to the Mixture-Target condition 76% (72–80%; −9%).

Lead vocals performed best with an accuracy of 96% (93–99%) and showed the smallest decrease by changing the order (TBP: −1%, TBS: −2%) or removing the isolation by changing the filtering (Target-Mixture: −4%, Mixture-Target: −5%). This was followed by the guitar with an accuracy of 84% (80–86%), which in contrast to the vocals and pianos, achieved higher accuracies in the Target-Mixture TBS than in the Mixture-Target TBP condition and almost as well in the Mixture-Target TBP and the Mixture-Target TBS conditions (difference by order TBP: −17%, TBS: −7%. Difference by filter Target-Mixture: −11%, Mixture-Target: −1%). The piano with an accuracy of 79% (75–83%), showed a similar pattern as for the lead vocals and was generally better when it was isolated than when the isolation was lifted (difference by order TBP: −15%, TBS: −15%. Difference by filter Target-Mixture: −18%, Mixture-Target: −18%). This dependence on instruments was also corroborated by our model (Instrument: *χ*^2^ = 42.177, *p* < 0.001).

Compared to the unmodified stimuli in the first experiment, applying a bandpass filter to the target improved the detection of instruments for both presentation orders by up to 16%. Specifically, this improvement raised the accuracies in the Target-Mixture condition to 99% (Experiment 1: 88%) for the lead vocals, 95% for the guitar, and 95% for the piano (Experiment 1: 79%). This indicates that whereas the frequency content of the instrument signals was narrowed down to an octave band and isolated, the additional selective attention in the Target-Mixture condition may have acted as searchlight, allowing for the detection of the target with an improved accuracy. However, whereas the overall accuracy was generally higher compared to the first experiment, the gaps between the accuracy in the Target-Mixture and the Mixture-Target conditions were larger than before for all instruments except the lead vocals. This gap was smallest and almost non-existent for the lead vocals (Experiment 1: −2%, Experiment 2 TBS: −2%), and enhanced for the guitar (in comparison to the average of non-bass instruments in Experiment 1: −10%, Experiment 2 TBS: −17%) and the piano (Experiment 1: −8%, Experiment 2 TBS: −15%). An instrument specific deterioration was underpinned by our model, which revealed a notable smaller contribution of the order alone (Order: *χ*^2^ = 3.5474, *p* = 0.060) and a much stronger contribution for the interaction between instruments and presentation order (Interaction: *χ*^2^ = 8.3447, *p* < 0.015).

Relative to Experiment 1, the increased effect of presentation order in Experiment 2 could be interpreted as related to the narrowband nature of the target signals, as it was already discussed for the bass in the first experiment. In a global mode of listening, listeners are required to distribute attention across the whole musical scene, which may make it easier to miss narrowband signals in a mixture of wideband signals, or not to perceive them as individual signals. In contrast to the bass in Experiment 1, instruments in Experiment 2 occurred in frequency ranges in which the human hearing is particularly sensitive, which in turn still led to generally high detection accuracy. Here, the lead vocals also showed advantages over other instruments, which suggest that other characteristics of the lead vocals can be detected within the narrow band, leading to better detection accuracy.

Detection accuracies additionally dropped in all target categories and for both presentation orders when the passband-filter was applied to an accompaniment instrument rather than the target. Again, the lead vocals were by far the least affected target category, showing that the lead vocal salience remains prominent even when the voice is suppressed in frequency regions where it is usually mixed louder than the mix. The general deterioration for all instruments and orders could be the by-product of a strategy in which participants listened primarily to the octave bands in which two-thirds of the targets appeared. Another reason for this pattern of results could be that the target did no longer occur in single octave band and thus targets were again subject to masking. A further possibility would be that the passband used here covers a particularly sensitive frequency region of human hearing, so that sound events in this area may be particularly salient.

Taken together, the spectral filtering guaranteed that the target stood out from the mixture, hence resulting in high detection accuracies in the Target-Mixture condition. In the Mixture-Target condition, accuracies were distinctly lower. As in the first experiment, the lead voice showed by far the smallest difference between the different orders of presentation. When the isolation of the target was removed, all instruments showed a deterioration of detection accuracies. Again, the lead vocals achieved the highest accuracy compared to the other instruments, yet with a smaller deterioration across presentation orders. Thus, an explanation of the lead vocal salience does not seem to be due to less susceptibility to masking in frequency regions in which the vocals are mixed with higher levels than the sum of the accompanying instruments.

## Experiment 3: Sound Level Equalization

Motivated by the relatively high sound levels of the lead vocals, here, we aimed to manipulate the level ratios of targets relative to the accompaniment to investigate whether this manipulation would affect detection performance and the observed differences between the presentation orders. As target categories, we selected the bass and lead vocals categories, because both were shown to be the conditions with lowest and highest performance in the first experiment, respectively. Since both target categories differ greatly in their spectral components, with bass being present mainly in the low frequencies and lead vocals in the mid and high frequencies, listeners could adopt a strategy where they would only listen to one of the distinct spectral regions. To avoid this, we added an additional experimental condition that contained instruments from all other target categories.

### Participants

A total of 55 participants with a mean age of 24.4 years (SD = 5.1, range = 18–33) were tested in the experiment. A total of 20 out of 27 participants passed the headphone screening for the Target-Mixture condition and 20 out of 28 for the Mixture-Target condition (age = 24, SD = 3.5, range: 19–33). Nine participants in the Target-Mixture condition and seven participants in the Mixture-Target condition described themselves as either amateur or professional musicians. Only participants passing the headphone screening were included in the analysis.

### Stimuli and Procedure

The target categories lead vocals, bass, and individual instruments from the categories drums, guitar, piano, synthesizer, strings, and winds were chosen as targets for the instrument conditions. Excerpts with targets from lead vocals, bass, and the mixed category appeared equally often. The sound level ratio between the targets and mixtures was set to one of three possible levels where the broadband level of the target was either 5, 10, or 15 dB below the level of the mixture (referred here as −5, −10, and −15 dB condition). To accomplish this, the 2-s instrument and mixture signal were separately analyzed using a 100 ms sliding window. For every window, the A-weighted sound level was computed using the *weightingFilter* function in Matlab (Audio Toolbox Version 2.1, MathWorks Inc., Natick, MA, United States) followed by a sound level estimation *via* RMS calculation. We normalized the average sound levels of each stimulus to −15 dBFS.

The headphone screening test was based on Milne et al. (2020). In the training phase of the main experiment, one excerpt with and without a target were presented for each of the three target categories and for each of the three sound level ratio conditions, summing up to 18 stimuli in total. In the experimental phase of the main experiment, 180 stimuli were presented, divided into 60 stimuli for each of the three target categories and further subdivided into 20 stimuli for each sound level conditions. The average duration of the experiment was 35 min.

### Results and Discussion

**Figure 6:**
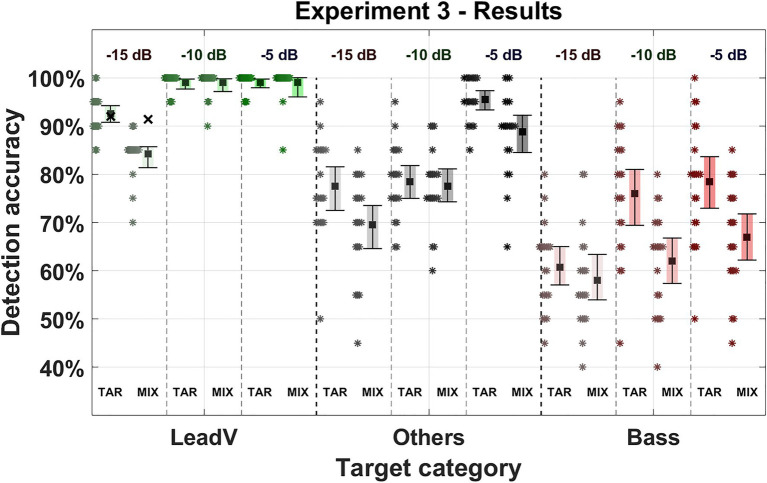
Detection accuracy in Experiment 3. Three instrument and vocal categories were used as targets (lead vocals, bass, and others = drums, guitar, piano, strings, synthesizer, and winds). The sound level ratio between the target and mixture was adjusted to either −5, −10, and −15 dB and is listed in the upper area of the figure, decreasing from right to left. The Square marks the mean detection accuracy for a given target category. Error bars indicate 95% CIs. Asterisks represent data from individual participants for the given target category. “TAR” denotes the presentation order “Target-Mixture” where the target cue was presented followed by a mixture. “MIX” denotes the presentation order “Mixture-Target” where a mixture was presented followed by the target cue. The green cross above the lead voice in the −15 dB condition marks the averaged detection accuracy when all stimuli that were consistently answered incorrectly were excluded (for details, see Results).

Results for the third experiment are shown in Figure 6 (for details, see Supplementary Tables 5, 6). Changes in the sound level ratio, presentation order, and target category affected the detection accuracy. The best performing condition was the lead vocal target category with a level ratio of −5 dB with an averaged accuracy of 99% (98–100%) in the Target-Mixture condition and 100% (100–100%) in the Mixture-Target order. Lowest detection accuracy was achieved by the lowest level ratio of −15 dB in the bass category ranging from 60% (57–64%) in the Target-Mixture condition to 58% (53–63%) in the Mixture-Target order. Within the same presentation order, the Target-Mixture condition achieved a generally higher accuracy, whereas the mean accuracy of all categories in the Mixture-Target condition deteriorated from 83% (82–84%) to 78% (75–80%; −5%).

Averaged across all sound level ratios, the lead vocals showed the highest detection accuracy and was most unaffected by a change in presentation order showing a slightly better accuracy of 97% (96–98%) in the Target-Mixture condition compared to the Mixture-Target condition with an accuracy of 95% (93–96%; −2%). In the category of multiple instruments, the detection accuracy deteriorated from 84% (81–86%) in the Target-Mixture condition to 79% (76–82%; −5%) in the Mixture-Target condition. The bass achieved the overall lowest accuracy dropping from 71% (68–74%) in the Target-Mixture condition to 63% (60–66%; −8%) in the Mixture-Target condition. These results are similar to the results of Experiment 1, where the lead vocals performed best while the bass performed worst.

In summary, contrary to our assumptions using higher sound levels and equalizing the levels had not resulted in a cancellation of the order influence, as it was still present in most conditions but not present for the lead vocals. This reasoning was further supported by the statistical model, which showed relevant interaction between the instruments and presentation orders for the third experiment (Interaction: *χ*^2^ = 1.257, *p* < 0.001). However, if we draw a comparison between Experiments 1 and 3, the effect of presentation order was reduced considerably (Bass: Exp.1 = −19%, Exp.3 = −10%. Other instruments: Exp.1 = −12%, Exp.3 = −5%).

Similar to the first and second experiment, the lead vocals stood out and achieved the highest accuracy. A decline in accuracy of 7% in the Target-Mixture and a considerably larger decline of 15% in the Mixture-Target conditions could only be observed in the lowest sound level condition. An observation of the individual sound levels shows a clear difference between both presentation orders in the lowest level condition: −0% (−5 dB), −0% (−10 dB), and −8% (−15 dB). Yet, a closer look at individual stimuli revealed that this decrease was based on a few distinct stimuli that achieved low detection accuracies (for a detailed view, see Supplementary Figures 3, 4). In the Target-Mixture condition, 17 out of 20 stimuli exceeded 90% detection accuracies, while one stimulus was close to chance level at 56%, whereas two stimuli were almost collectively answered incorrectly, achieving an accuracy of only 15%. This agreement was even stronger in the Mixture-Target condition where 15 out of 20 stimuli achieved 100% accuracy, one stimulus achieved 95% accuracy, and the last four stimuli achieved an accuracy of less than 16%. When we excluded all stimuli that were consistently answered incorrectly (detection accuracy of 0%), the results remained identical for all conditions except for the lead vocals in the lowest level ratio. Here, accuracy in the Target-Mixture condition remained at 92% (+0%) and in the Mixture-Target condition from 86 to 91% (+5%), almost closing the gap between the two presentation orders that arose in the −15 dB condition (from an order effect of 6–3%), although we only conservatively screened out stimuli that were consistently answered incorrectly by all participants (for a detailed view, see Supplementary Table 5). For these reasons, a generalization of the results of the lead vocals at the lowest level ratios seems questionable, because accuracies here seem to be mainly driven by a few stimuli rather than the systematic change in level ratio.

The target category “others” was most affected by a level decrease, declining by 18% in both presentation orders. Differences between presentation orders in this target category varied at different levels: −7% (−5 dB), −1% (−10 dB), and −8% (−15 dB). At the −10 dB condition revealed an ambiguous result, where the difference between the two presentation orders is only marginal. Considering all seven remaining conditions, which show clear effects, we interpret the present pattern of results as indication that the adjustment of the sound level ratios did not eliminate the order effect for the instruments and did not cause any robust order effect for the lead vocals.

The bass was slightly less affected by a decrease in level, achieving 18% in the Target-Mixture and 9% in the Mixture-Target conditions. With decreasing level, a consistently deteriorating detection of the mixture-target condition could be observed: −12% (−5 dB), −14% (−10 dB), and −3% (−15 dB).

In summary, by varying the target-to-accompaniment level ratio, we here observed effects of presentation order at different level ratios for a mixed category of instruments and for the bass instrument but no notable effect for the lead vocals. This once more confirmed the inherent salience of lead vocals in musical mixtures, which seems to be stable across sound levels.

## General Discussion

In this study, we aimed to investigate ASA for musical instruments and singing voices and its modulation by selective auditory attention. Excerpts of popular music were presented in an instrument and singing voice detection task. Participants listened to a 2-s except either globally with a mixture preceding a target cue or selectively with a target cue preceding the mixture. We hypothesized that listeners’ performance would be facilitated when a target cue is presented prior to the presentation of the mixture. In addition, we suspected a detection advantage of lead vocals relative to other instruments.

In line with our assumptions regarding the presentation order and previous studies (e.g., Bey and McAdams, 2002; Janata et al., 2002), detection performance was best when the target cue was presented before the mixture, highlighting the role of endogenous top-down processing to direct selective auditory attention. Accuracy worsened when listeners were presented the target after the mixture. This was the case for all target categories, apart from the lead vocals, which not only achieved the best detection accuracies among all target categories, but also showed no (or clearly much smaller) decreases of detection accuracies across the two orders of presentation. Although we initially assumed higher detection accuracy for the lead vocals, the latter finding exceeded our expectations about vocal salience in musical mixtures.

In a second and third experiment, we investigated how manipulations of acoustical features would affect lead vocal salience by eliminating differences between the target categories in relative sound level or release from spectral masking. However, contrary to our hypothesis, even when targets were completely unmasked from the mixture, or when the same sound levels were applied, lead vocals retained a unique role and robustly achieved the highest detection accuracies results across all manipulations, with a clear advantage over all other instruments. These findings support a unique role of the lead vocals in musical scene perception. More generally, this pattern of results is consistent with previous work in which singing voices have been shown to be perceptually privileged compared to other musical instruments by yielding faster processing (Agus et al., 2012) and more precise recognition rates (e.g., Suied et al., 2014; Isnard et al., 2019) as well as a stronger cortical representation (e.g., Levy et al., 2001) compared to other instruments. Our results demonstrate that auditory attention is drawn to the lead vocals in a mix, which complements knowledge about pre-attentive perceptual biases in musical scene analysis such as the high-voices superiority effect (Trainor et al., 2014).

From a music production point of view, it may be argued that the facilitated detection of lead vocals could be a result of acoustic cues that arise from common tools such as compression and notch filtering, which allow the vocals to “come through” and be perceived as the most prominent sound “in front of” the mixture. The results of Experiments 2 and 3 render this hypothesis unlikely, however. Despite complete unmasking of target categories in Experiment 2 and drastic changes of level in Experiment 3, the lead vocals remained the only target category that did not show an order effect and hence may be interpreted as the only target category with specific auditory salience.

In several recent studies, Weiss and colleagues provided evidence for a memory advantage of vocal melodies compared to melodies played by non-vocal musical instruments. Analyses of the recognition ratings for old and new melodies revealed that listeners more confidently and correctly recognized vocal compared to instrumental melodies (Weiss et al., 2012). It was further shown that the presentation of vocal melodies, as well as previously encountered melodies, was accompanied by an increase in pupil dilation (Weiss et al., 2016), often indirectly interpreted as indicator of raised engagement and recruitment of attentional resources. Our results directly highlight that those vocal melodies appear to act as a type of robust attentional attractors in musical mixtures, hence providing converging evidence for a privileged role of voices in ASA.

The lead vocal salience observed here could be due to a human specialization to process speech sounds. Therefore, lead vocals may have benefited from their speech features. Previous studies have demonstrated that phonological sounds, such as words and pseudo-words, are easier to detect than non-phonological complex sounds (Signoret et al., 2011). Therefore, an idea worth exploring is whether the advantage of lead vocals is still present when the vocal melody is sung with non-phonological speech, sung by humming, or played by an instrument. Another speech-like aspect that could make vocals more salient is the semantic content of the lyrics. Trying to grasp the meaning behind the lyrics could therefore draw attention to the vocals. Although test participants were not English native speakers, in Germany, it is common to listen to songs with English lyrics.

Another origin for the lead vocal salience could lie on a compositional level. In the used excerpts of popular music, lead voices certainly acted as the melodic center of the songs. The resulting melodic salience is known to dominate the perception of a musical scene (Ragert et al., 2014). A question which would be interesting to examine is whether the vocal salience found here would also be found if the main melody were played by another instrument and whether in this case the instrument would show enhanced auditory salience.

## Conclusion

We used short excerpts of popular music in a detection task to investigate the influence of selective auditory attention in the perception of instruments and singing voices. Participants were either directed to a cued target vocal or instrument in a musical scene or had to listen globally in the scene before the cued target was presented. As expected, in the presentation order where no cue was given before the mixture and thus no additional support for endogenous top-down processing was provided, detection accuracy deteriorated. Whereas all instruments were affected by a change in the presentation order, the lead vocals were robustly detected and achieved the best detection accuracies among all target categories. To control for potential spectral and level effects, we filtered the target signals so that they were unmasked in a particular frequency band and eliminated sound levels differences between the targets. This facilitated instrument detection for the presentation order where the target was presented first, but not for the order where the mixture was presented first. These results indicate that the observed lead vocal salience is not based on acoustic cues in frequency region where the lead vocals are mixed at higher levels than the sum of accompanying instruments. It was further found that higher sound levels resulted in more similar scores across the presentations orders, but there remained clear order effect for all instruments except for the lead vocals, suggesting that the higher level-ratios of vocals are not the origin of the lead vocal salience. This confirms previous studies on vocal significance in ASA. Further research is needed to assess whether these features are based on its unique vocal qualities, semantic aspects of the vocal signal, or on the role of the center melody in musical mixtures.

## Data Availability Statement

The raw data supporting the conclusions of this article will be made available by the authors, without undue reservation.

## Ethics Statement

The studies involving human participants were reviewed and approved by Kommission für Forschungsfolgenabschätzung und Ethik. The patients/participants provided their written informed consent to participate in this study.

## Author Contributions

MB and KS designed the study. LP provided the stimuli. MB collected and analyzed the data and wrote a first draft of the manuscript. KS and LP revised the manuscript. All authors contributed to the article and approved the submitted version.

## Funding

This research was supported by a Freigeist Fellowship of the Volkswagen Foundation to KS.

## Conflict of Interest

The authors declare that the research was conducted in the absence of any commercial or financial relationships that could be construed as a potential conflict of interest.

## Publisher’s Note

All claims expressed in this article are solely those of the authors and do not necessarily represent those of their affiliated organizations, or those of the publisher, the editors and the reviewers. Any product that may be evaluated in this article, or claim that may be made by its manufacturer, is not guaranteed or endorsed by the publisher.
