# Supplementary material for: Listening in the Mix: Lead Vocals Robustly Attract Auditory Attention in Popular Music
Source: Front Psychol. 2021 Dec 23;12:769663. doi: 10.3389/fpsyg.2021.769663 (PMC8744650; doi:10.3389/fpsyg.2021.769663)
Supplement: Supplementary file 1 [file Data_Sheet_1.PDF]

Listening in the mix: Lead vocals robustly attract auditory attention in popular music

Supplementary Material

1 Detection accuracies and analysis results

1.1 Experiment 1 - Detection Accuracies

Table 1: Experiment 1 - Detection accuracies in the first experiment. Accuracies for the detection of a target vocal or instrument in a mixture of multiple instruments are listed by target category and order of target presentation. In the Target-Mixture order a single target vocal or instrument was presented followed by a mixture of multiple instruments. In the Mixture-Target order a mixture of multiple instruments was presented followed by a single target vocal or instrument. The square brackets contain 95% confidence intervals.

**Target category**	**Target-Mixture: Average detection accuracy in %**	**Mixture-Target: Average detection accuracy in %**
Lead Vocals	88 [85 - 91]	86 [80 - 89]
Drums	85 [82 - 88]	74 [72 - 78]
Synthesizer	86 [80 - 86]	75 [72 - 78]
Piano	79 [76 - 83]	71 [68 - 74]
Bass	81 [76 - 86]	62 [59 - 66]

1.2 Experiment 1 - GLME Factors

Table 2: Experiment 1 – Analysis of GLME Factors. The contribution of factors used within the generalized linear mixed effect model and their interactions were analysed via an ANOVA. Order refers to the influence of the cue presentation that was presented either before or after the mix. Instrument refers to the differences between the instrument categories. Colons indicate the interaction of the respective factors.

**Factor**	***DF***	***Sum Sq***	***Mean Sq***	***χ^2 value***	***p val***
Order	1	39.634	39.634	38.878	<0.001
Instrument	4	97.971	24.493	97.881	<0.001
Order:Instrument	4	13.063	3.266	13.059	0.011

1.3 Experiment 2 - Detection Accuracies

Table 3: Experiment 2 - Detection accuracies. Accuracies for the detection of a target vocal or instrument in a mixture of instruments are listed by target category, filter typ and order of target presentation. The filter denotes the used filtering and is either T_{BP} when a bandpass was applied to the target suppressing frequencies outside of an octave band (1 – 2 kHz, 2 – 4 kHz) or T_{BS} when a bandstop was applied to the target suppressing frequencies in only in an octave band (1 – 2 kHz, 2 – 4 kHz). In the Target-Mixture order a single target vocal or instrument was presented followed by a mixture of instruments. In the Mixture-Target order a mixture of instruments was presented followed by a single target vocal or instrument. The square brackets contain 95% confidence intervals.

**Target category**	**Filter**	**Target-Mixture: Average detection accuracy in %**	**Mixture-Target: Average detection accuracy in %**
Lead vocals	T_{BP}	99 [97 - 100]	98 [97 - 99]
Lead vocals	T_{BS}	95 [92 - 96]	93 [90 - 94]
Guitar	T_{BP}	95 [93 - 97]	78 [75 - 82]
Guitar	T_{BS}	84 [80 - 87]	77 [72 - 80]
Piano	T_{BP}	95 [93 - 97]	80 [75 - 85]
Piano	T_{BS}	77 [72 - 81]	62 [59 - 66]

1.4 Experiment 2 – GLME Factors

Table 4: Experiment 2 – Analysis of GLME Factors. The contribution of factors used within the generalized linear mixed effect model and their interactions were analysed via an ANOVA. Order refers to the influence of the cue presentation that was presented either before or after the mix. Filter refers to the differences between the passband filtering of either the target or a randomly drawn accompanying instrument. Instrument refers to the differences between the instrument categories. Colons indicate the interaction of the respective factors.

**Factor**	***DF***	***Sum Sq***	***Mean Sq***	χ^2 ***value***	***p val***
Order	1	3.390	3.3900	3.547	0.060
Filter	1	18.560	18.560	18.657	<0.001
Instrument	2	41.447	20.723	42.177	<0.001
Order:Filter	2	0.016	0.016	0.016	0.901
Order:Instrument	2	8.250	4.125	8.345	0.015
Filter:Instrument	2	11.699	5.850	11.689	0.003
Order:Filter: Instrument	2	2.070	1.035	2.067	0.355

1.5 Experiment 3 - Detection Accuracies

Table 5: Experiment 3 - Detection accuracies. Accuracies for the detection of a target vocal or instrument in a mixture of instruments are listed by target category, sound level ratio and order of target presentation. The asterisk shows the accuracy when all stimuli with a detection rate of 0% are excluded (for further explanation see results). The sound level ratio indicates the sound level difference between the target and the mixture. In the Target-Mixture order a single target vocal or instrument was presented followed by a mixture of instruments. In the Mixture-Target order a mixture of instruments was presented followed by a single target vocal or instrument. The square brackets contain 95% confidence intervals.

**Target category**	**Sound level ratio**	**Target-Mixture: Average detection accuracy in %**	**Mixture-Target: Average detection accuracy in %**
Lead vocals	-5	99 [98-100]	99 [100-100]
Lead vocals	-10	99 [98-100]	99 [97-100]
Lead vocals	-15	92 [91-95]	84 [82-86]
**Lead vocals***	**-15***	**92 [91-96]***	**91 [89-94]***
Other	-5	96 [93-97]	89 [84-92]
Other	-10	79 [75-82]	78 [74-81]
Other	-15	78 [72-81]	70 [64-73]
Bass	-5	79 [73-83]	67 [62-71]
Bass	-10	76 [70-81]	62 [57-66]
Bass	-15	61 [57-65]	58 [53-63]

1.6 Experiment 3 - GLME Factors

Table 6: Experiment 3 – Analysis of GLME Factors. The contribution of factors used within the generalized linear mixed effect model and their interactions were analysed via an ANOVA. Order refers to the influence of the cue presentation that was presented either before or after the mix. Ratio refers to the differences between the three used levels ratios between the target and mixture. Instrument refers to the differences between the instrument categories. Colons indicate the interaction of the respective factors.

**Factor**	***DF***	***Sum Sq***	***Mean Sq***	**χ^2 *value***	***p val***
Order	1	15.12	15.122	15.121	<0.001
Ratio	2	165.89	82.945	82.944	<0.001
Instrument	2	319.29	159.644	159.644	<0.001
Order:Ratio	1	0.142	0.142	0.339	0.560
Order:Instrument	2	2.51	1.257	1.257	<0.001
Ratio:Instrument	4	128.26	32.065	32.065	0.539
Order:Ratio:Instrument	4	14.312	3.578	3.467	0.008

2 Pilot Results

(A)

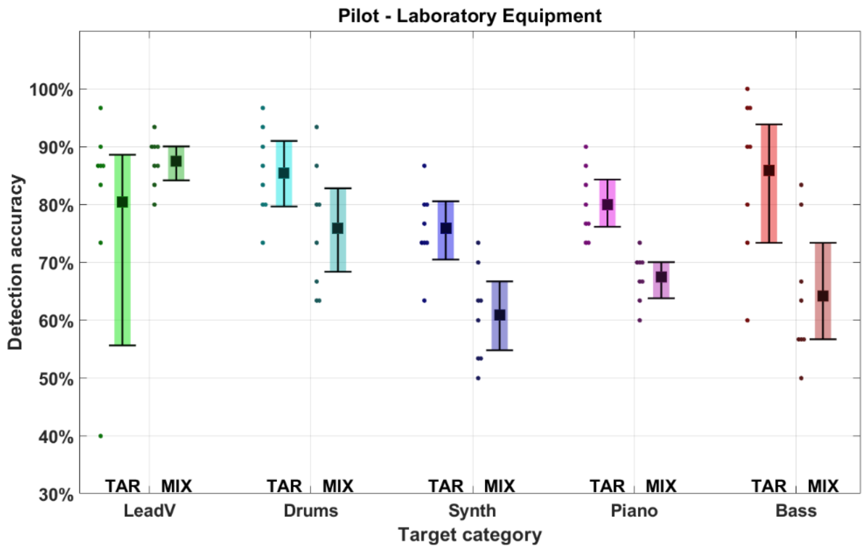

(B)

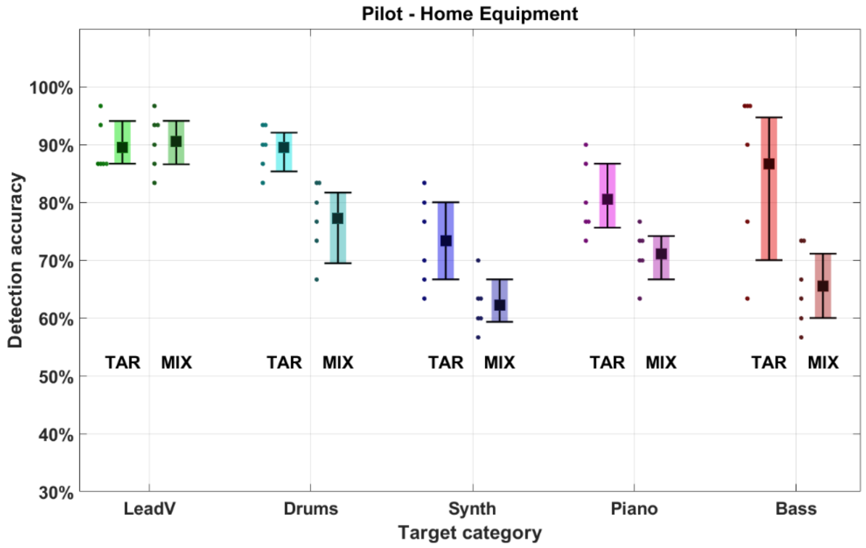

Supplementary Figure 1. Detection accuracy in pilot experiment: A pilot experiment that was similar to the first experiment was conducted using two different hardware setups. (A) Participants used the same equipment which was calibrated in sound level and conducted the experiment. (B) Participants used their personal uncalibrated equipment and conducted the experiment. Results showed similar results for both setups. Five instrument categories were used as targets (lead vocals, drums, synthesizer, piano, bass). The square marks the mean detection accuracy for a given instrument category. Error bars indicate 95% confidence intervals. Left-pointing triangles represent the average accuracy of an individual participant (n_{LAB} = 8, n_{HOME} = 7) for the given instrument category. “TAR” denotes the presentation order “Target-Mixture” where the target cue was presented followed by a mixture. “MIX” denotes the presentation order “Mixture-Target” where a mixture was presented followed by the target cue.

3 Experiment 1 – Fatigue

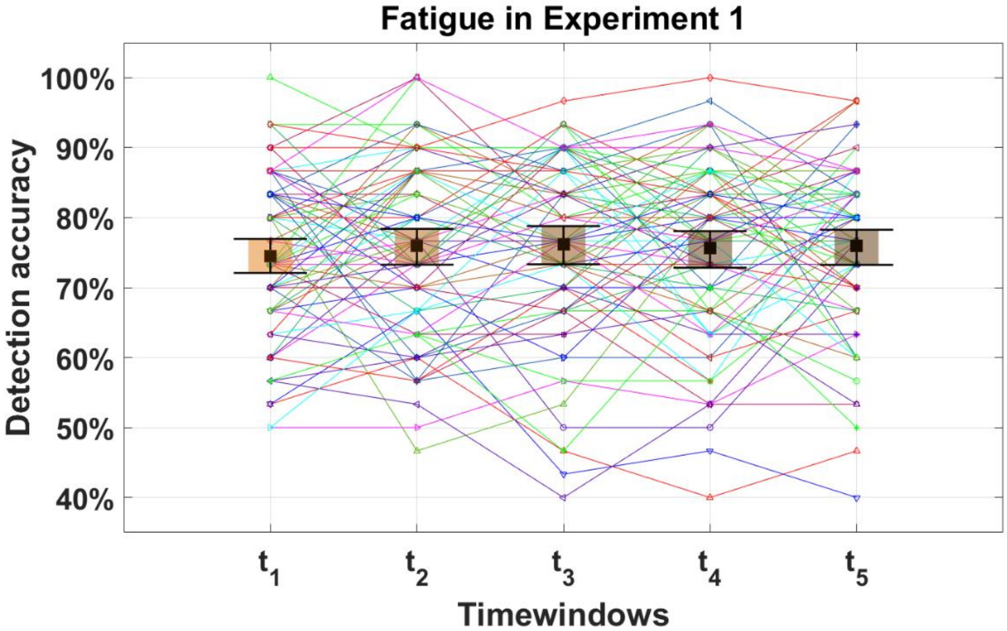

Supplementary Figure 2: Experiment 1 – Fatigue: Change of accuracy over the duration of Experiment 1. Experiment was divided into time windows "**t**", each containing 30 subsequent stimuli. The square marks the mean accuracy for a given time window. Error bars indicate 95% confidence intervals. Individual participant data is represented with a colored marker and connect by a line between time windows. Time window "$\mathbf{t_1}$" contains the first 30 stimuli while "$\mathbf{t_5}$" contains the last 30 stimuli.

4 Experiment 3 - Consistency of responses

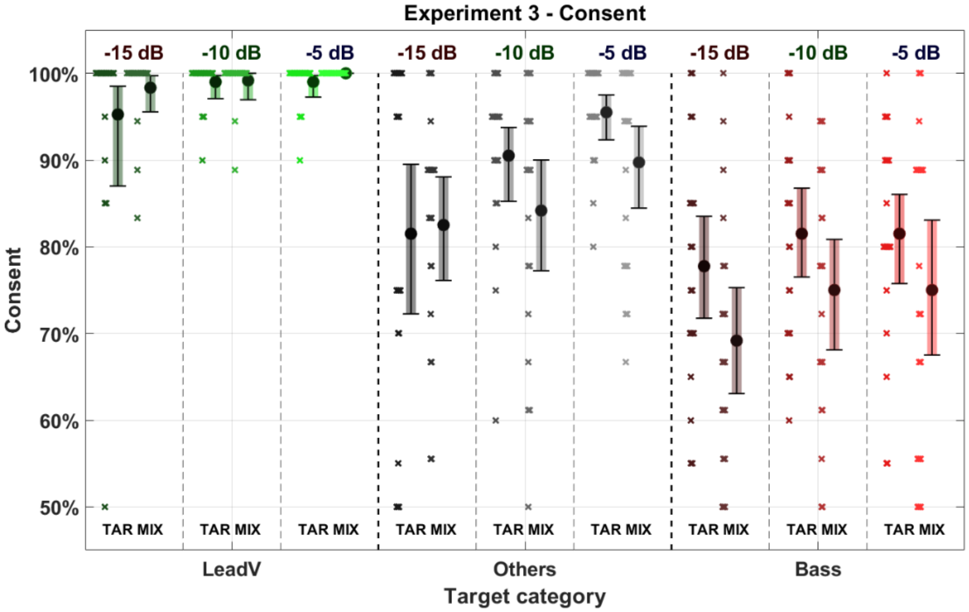

Supplementary Figure 3: Experiment 3 – Consent: Consistency in the responses of the participants for each stimuli of the given target category. For each stimulus, the distribution of responses was determined. A 100% consent means that all participants consistently answered the stimuli either incorrectly or correctly, while 50% indicates the greatest disagreement among participants. The sound level ratio between the target and mixture was adjusted to either -5 dB, -10 dB, -15 dB and is listed in the upper area of the figure, decreasing from right to left. The circle marks the mean consent for a given instrument category. Error bars indicate 95% confidence intervals. Crosses represent the average consent of an individual stimulus. “TAR” denotes the presentation order “Target-Mixture” where the target cue was presented followed by a mixture. “MIX” denotes the presentation order “Mixture-Target” where a mixture was presented followed by the target cue.

5 Experiment 3 – Item Score

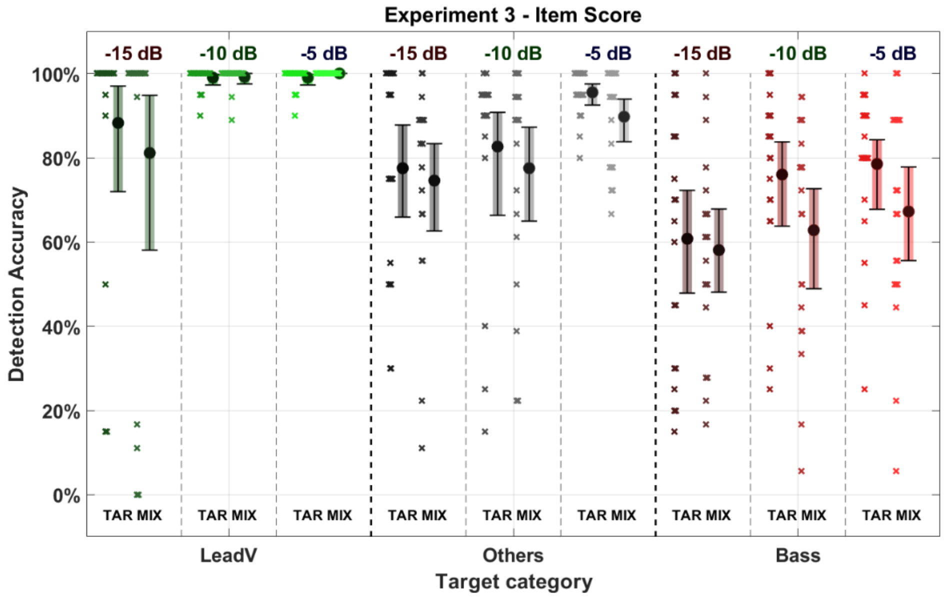

Supplementary Figure 4: Experiment 3 – Item Score: Detection accuracy for each stimuli of the given target category. The sound level ratio between the target and mixture was adjusted to either -5 dB, -10 dB, -15 dB and is listed in the upper area of the figure, decreasing from right to left. The circle marks the mean accuracy for a given instrument category. Error bars indicate 95% confidence intervals. Crosses represent the average accuracy of an individual stimulus. “TAR” denotes the presentation order “Target-Mixture” where the target cue was presented followed by a mixture. “MIX” denotes the presentation order “Mixture-Target” where a mixture was presented followed by the target cue.
